# The Effect of Oxygen Therapy on Oxidative Stress Index in Patients with Acute Myocardial Infarction; a Letter to the Editor

**Published:** 2019-01-01

**Authors:** Afshin Amini, Abbas Mahdavipour

**Affiliations:** 1. Emergency Department, Imam Hossein Hospital, Shahid Beheshti University Of Medical Sciences, Tehran, Iran.

**Keywords:** Oxygen Inhalation Therapy, Oxidative Stress, Inferior Wall Myocardial Infarction, Anterior Wall Myocardial Infarction

Tissue hypoxia is a key factor for cell death after acute myocardial infarction (MI). It seems that increase in the relative oxygen pressure in inhaled air can be an effective treatment option for treating acute MI. However, contradicting findings and results have been published regarding using oxygen therapy in patients with acute MI (1, 2). Some researchers have believed that generation of free radicals, induction of oxidative stress, and damage to cell membrane are among side effects of O_2_ consumption (3, 4). It has been shown that O_2_ therapy can increase microvascular resistance, result in a decrease in coronary blood flow and cardiac output, and bring about numerous negative effects such as increase in the risk of arrhythmia and cellular damage (4).

In aerobic biological systems, defense mechanisms have been put in place for fighting free radicals and reactive oxygen species to neutralize or minimize the damaging effects of these invasive factors. Some components of the defense system are enzymes such as superoxide dismutase, glutathione peroxidase, catalase, and…, which are synthesized in the body; yet, some of the other components of this system such as vitamin E, beta carotene, and etc. should be provided through the diet (5, 6). 

During reperfusion, hypoxanthine that had aggregated in the ischemia phase, is metabolized into xanthine via xanthine oxidase enzyme and in this process, superoxide radicals are created and changed into hydrogen peroxide (H_2_O_2_) or hydroxyl radicals (OH). Oxidative damage happens when antioxidant defense consisting of glutathione, and antioxidant enzymes (like superoxide dismutase, glutathione peroxidase, catalase, and …) is broken down (7).

It seems that measurement of oxidative stress biomarkers can be used as an index for evaluating the effect of oxygen therapy in patients with acute MI. Therefore, for testing this hypothesis, in a randomized clinical trial, the researchers of the present study studied 90 patients with acute MI, who had presented to the emergency department of Imam Hossein Hospital within less than 12 hours from the initiation of their symptoms. Initially venous blood was drawn from all the patients for measuring oxidative stress parameters. Then patients were randomly divided into 2 groups using block randomization. The first group was continuously treated with 6 to 8 liters of oxygen per minute via facial mask and without considering blood O2 saturation, and the second group only received oxygen if their arterial blood saturation level dropped to less than 94%. At the end of the 6^th^ hour after hospitalization blood was drawn from all of the patients (for the second time) to assess oxidative stress parameters including total oxidant enzymes. Patients with a history of MI, known cardiac and respiratory diseases (such as obstructive pulmonary disease or obstructive sleep apnea), unstable vital signs, endocrine disorders, hematologic abnormalities, autoimmune diseases or malignancies, pregnancy, and those who did not give consent for participation in the study were excluded.

The results showed that case and control groups were not significantly different regarding mean age (60.67 ± 9.17 vs. 61.16 ± 7.53 years; p = 0.783), sex (68.9% male vs. 64.4%; p = 0.823), and oxidant level on admission (p = 0.104).

Oxidant levels in the control (156.41 ±15.94 vs. 141.84 ± 15.80 µmol/L; p <0.0001) and case (150.94 ± 15.69 vs. 142.59 ±15.35 µmol/L;‌ p <0.0001) groups had significantly reduced after 6 hours. However, the oxidative stress indices in the control group that received oxygen selectively had decreased significantly more than the case group (p <0.0001).

In 2012, Ranchord et al. showed that there is no evidence regarding high oxygen concentrations being beneficial or harmful in comparison with titrated oxygen in patients with MI (8). The study by Tatarkova et al. showed that normobaric oxygen therapy will be associated with oxidative damage of myocardia and weakening of antioxidant defense (9). 

Observations of the authors of this study showed that the rate of oxidative indices in patients with acute MI who had received oxygen in a titrated manner and only if their arterial blood oxygen saturation had dropped to less than 94% had decreased more. Therefore, it seems that oxygen should literally be seen as a drug and should be prescribed for the patients only when necessary and its benefits outnumber its harms.

It seems that giving excess oxygen to acute MI patients who have normal Spo2 should be avoided. Of course, there is need for more comprehensive studies to be able to comment with certainty in this regard.

## References

[1] Beasley R, Weatherall M, Aldington S, Mchaffie D, Robinson G (2007). Oxygen therapy in myocardial infarction: an historical perspective. Journal of the Royal Society of Medicine.

[2] Lorgis L, Zeller M, Dentan G, Sicard P, Richard C, Buffet P (2010). The free oxygen radicals test (FORT) to assess circulating oxidative stress in patients with acute myocardial infarction. Atherosclerosis.

[3] Hammond B, Hess ML (1985). The oxygen free radical system: potential mediator of myocardial injury. Journal of the American College of Cardiology.

[4] Weston C (2010). Oxygen therapy in acute myocardial infarction–too much of a good thing?. Cochrane Database of Systematic Reviews.

[5] Gill R, Tsung A, Billiar T (2010). Linking oxidative stress to inflammation: Toll-like receptors. Free Radical Biology and Medicine.

[6] Sharma V, Shukla RK, Saxena N, Parmar D, Das M, Dhawan A (2009). DNA damaging potential of zinc oxide nanoparticles in human epidermal cells. Toxicology letters.

[7] Singhal AB, Benner T, Roccatagliata L, Koroshetz WJ, Schaefer PW, Lo EH (2005). A pilot study of normobaric oxygen therapy in acute ischemic stroke. Stroke.

[8] Ranchord AM, Argyle R, Beynon R, Perrin K, Sharma V, Weatherall M (2012). High-concentration versus titrated oxygen therapy in ST-elevation myocardial infarction: a pilot randomized controlled trial. American heart journal.

[9] Tatarkova Z, Engler I, Calkovska A, Mokra D, Drgova A, Kuka S (2012). Effect of normobaric oxygen treatment on oxidative stress and enzyme activities in guinea pig heart. General physiology and biophysics.

